# A Procedure for Extending Input Selection Algorithms to Low Quality Data in Modelling Problems with Application to the Automatic Grading of Uploaded Assignments

**DOI:** 10.1155/2014/468405

**Published:** 2014-07-07

**Authors:** José Otero, Ana Palacios, Rosario Suárez, Luis Junco, Inés Couso, Luciano Sánchez

**Affiliations:** ^1^Computer Science Department, Universidad de Oviedo, Sedes Departamentales, Edificio 1, Campus de Viesques, 33203 Gijón, Spain; ^2^Computer Science Department, Universidad de Granada, C/Periodista Daniel Saucedo Arana s/n, 18071 Granada, Spain; ^3^Statistics Department, E. U. I. T. Industrial, Universidad de Oviedo, Módulo 1, Planta 4, Campus de Viesques, 33203 Gijón, Spain

## Abstract

When selecting relevant inputs in modeling problems with low quality data, the ranking of the most informative inputs is also uncertain. In this paper, this issue is addressed through a new procedure that allows the extending of different crisp feature selection algorithms to vague data. The partial knowledge about the ordinal of each feature is modelled by means of a possibility distribution, and a ranking is hereby applied to sort these distributions. It will be shown that this technique makes the most use of the available information in some vague datasets. The approach is demonstrated in a real-world application. In the context of massive online computer science courses, methods are sought for automatically providing the student with a qualification through code metrics. Feature selection methods are used to find the metrics involved in the most meaningful predictions. In this study, 800 source code files, collected and revised by the authors in classroom Computer Science lectures taught between 2013 and 2014, are analyzed with the proposed technique, and the most relevant metrics for the automatic grading task are discussed.

## 1. Introduction

Online courses are ubiquitous nowadays. Almost every institution, university, college, or high-school offers freely accessible online courses. Massive Open Online Courses (MOOCs) and Distance Learning have a large impact in developing countries, helping to improve education in poor regions.

Learning Management Systems or Content Management Systems are used to provide the students with different kinds of material and also allow students and teachers to interact via lectures, assignments, exams, or gradings. However, the resources needed for tracking students and taking examination are time consuming for the organizing institutions; thus, there is demand for intelligent techniques that help the instructor to manage large groups of students. In particular, procedures that partially or completely automate the grading process are sought, understood as taking standardized measurements of varying levels of achievement in a course [1].

There are topics, however, whose qualification is troublesome. Think for instance of computer programming, where the usual examination procedure consists in challenging the students with a set of problems to be solved. In online courses, the student's solutions, comprising one or more source code files, are uploaded to the platform, where the person at charge scores the task. This needs a long time and it is also difficult for the teacher to be objective and unbiased. If the grading depends not only on the program output correctness (using a set of sample data inputs) but also on the structure of the solution (data types, control flow, ans efficiency) or the documentation quality, the situation is even worse. In addition to this, the students should follow the usual software developing process and thus the solution of each assignment should pass through several stages until it reaches a maturity level such that it can be submitted as a completed task. Those intermediate stages could provide a valuable feedback to the teacher, regarding individual students' needs and also teacher's lectures, materials or strategies quality.

### 1.1. Automatic Grading in Online Courses

The automatic grading is a problem that has been addressed by many researchers. To name some, in [2] a semiautomated system for task submission and grading is proposed, but the grading itself must be done manually by the teacher. The* WebToTeach* system [1], on the contrary, is able to check submitted source code automatically. Similar to this and focused on programming, the methods in [3] or [4] achieve an automatic grading by comparing the output of each student program with the output of a correct program. There is no measurement of the internals of the source code, which is labelled as correct if the output is correct, regardless of the solution strategy.

The* AutoLEP* system [5] is more recent. One of the salient points of this last work is a procedure to compare any implementation of an algorithm against a single model. Furthermore, in [6] a methodology is presented that accomplishes automatic grading by testing the program results against a predefined set of inputs and also by formally verifying the source code or by measuring the similarities between the control flow graph and the teacher's solution. The parameters of a linear model are found that averages the influence of the three techniques in order to match teacher's grade and automatic grading in a corpus of manually graded exercises. Finally, in [7], software metrics are used to measure the properties of the students' programs and a fuzzy rule-based system is used to determine how close the programs submitted by students and the solutions provided by the teacher are, partially achieving an automatic grading.

#### 1.1.1. Automatic Grading and Continuous Assessment

The former approaches pay particular attention to exam grading. In the problem at hand, this consists in comparing the outputs of student programs to those of a correct program, but there are secondary aspects about the internals of the source code (i.e., code style, documentation, etc.) that must be assessed too. In some cases, software metrics provide an additional insight [7].

However, the purpose of following an online course is arguably not to obtain a certificate but to acquire knowledge. From the instructor's side, it is important that an early corrective action is taken if learning difficulties are detected and therefore a continuous assessment of the student must be carried out. In this case, the picture is completely different. It is hard to combine MOOCs and continuous assessment; however, it is clear that the evolution of each student could not be tracked down to a single exam. Incremental measurements of the levels of achievement of each programming concept should be taken, with the help of the many different assignments that the students upload to the server hosting the course. The number and size of these assignments depend on the student, and some of its elements might be missing; not all the online students finish their tasks, and the amount of work carried out by the students is largely different.

Therefore, different sets of assignments must be combined and jointly considered by the grading system. The combination procedure must be resilient to missing data and incomplete assignments, as students will be graded on the basis of sets of data of different sizes. Finally, if software metrics are used to assess the quality of the assignments, not all of them are equally informative for each programming concept. Because of the mentioned reasons, in this paper,

- a method is proposed for building a fuzzy compound value that summarizes the values of the software metrics of different source files that are related to the same programming concept. This compound value takes into account both the average value and the dispersion of the different metrics;
- the relevance of the different metrics is assessed with an extension of a crisp feature selection algorithm to fuzzy data. It will be shown that the extension described in this paper exploits the available data in a real-world problem better than the alternatives;
- a learning fuzzy system that can extract if-then rules from interval and fuzzy data is used to build the rule based system that performs the grading on the basis of the metrics that are selected in the preceding step.

This paper is organized as follows: in Section 2, the method for combining the values of a metric over a set of different source files and a method for ranking the importance of the fuzzy aggregated values are described. In Section 3, the rule learning algorithm is described. In Section 4, numerical results are provided that validate the claims of this paper with actual data collected in classroom lectures in 2013 and 2014.  Section 5 concludes the paper and highlights future research lines.

## 2. Feature Selection for Regression with Vague Data

As mentioned, the grading process is intended to determine the level of achievement of each programming concept, which in turn is assessed by means of a set of source code files written by the students. The metrics of all files in these sets are jointly considered. Given that these sets are of different sizes for different students and some of its elements may be missing, a robust combination method is needed.

The proposed combination is based on the assumption that the application of a software metric to a given source code can be assimilated to the process of measuring the value of an observable variable or* item* that provides partial information to describe an unobservable or* latent* variable. In this case, the latent variable is the degree of assessment of a given programming concept. It is remarked that the information provided by different items may be in conflict.

The conversion of a set of items into a compound value that can be fed into a model has been solved in different ways in other contexts. For instance, in marketing problems, certain models have been designed where sets of items are preprocessed and aggregated into a characteristic value [8]. The most commonly used aggregation operator is the mean, although many different functions may be used instead [9].

In [10], however, a different approach was used: it was assumed that there exists a true value for the latent variable, but also that this value cannot be precised further than a set that contains it. In this respect, it is widely known that uncertainty in databases encompasses probabilistic and incomplete data, the former being a refinement of the latter, but some categories of imprecise data are hardly addressed in this framework, such as censored or interval-valued data. Imprecise probabilities-based representations are better suited for these problems, because the available knowledge about the possible values of the data by means of families of probability distributions.

**Figure 1:**
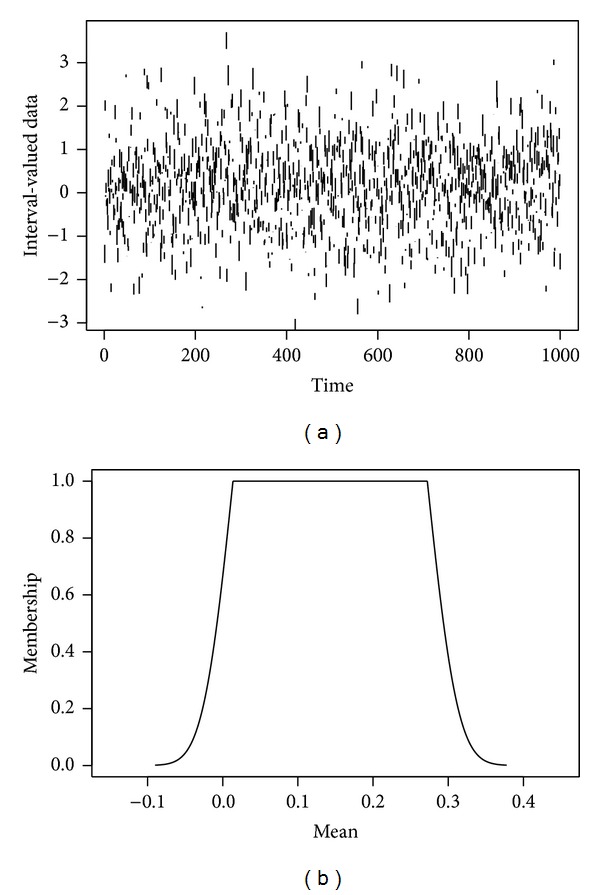
Fuzzy representation of LQD. (a) 1000 instances of an interval-valued database. (b) Fuzzy (possibilistic) representation of the mean value of the data in the left part. *α*-cuts of this fuzzy set are bootstrap-based confidence intervals of the interval valued data; that is, they are the smallest intervals containing at least a fraction 1 − *α* of the data.

Possibilistic representations are a particular case of these, general enough for modeling a wide range of practical problems: incomplete databases can be represented by means of the vacuous belief function (the set of all probability distributions) that models full ignorance, and other types of uncertain data, such as the aforementioned interval-valued or censored data can also be easily represented. Moreover, a possibilistic view of uncertainty is compatible with the use of fuzzy sets for describing partial knowledge about the data, because the contour function of a possibility distribution is a fuzzy set [11]. In this context, *α*-cuts of fuzzy sets may be linked to confidence intervals about the unknown value of the feature with significance levels 1 − *α* (see Figure 1 and reference [12]). This last property supports the use of intervals or fuzzy data for modelling the following types of uncertain or* low quality* data:

- unqualified sets of possible values, such as enumerations, {*x*_1_, *x*_2_,…, *x*_*N*_}, or interval-valued data, [*x*_1_, *x*_2_]. Note that missing data is represented by intervals spanning the whole domain of the variable. Censored data is represented in a similar way;
- qualified sets of possible values, where each item is associated with a probability value, an upper probability, or an interval of probabilities. These constructs often arise from the fuzzification interfaces of fuzzy rule-based systems. For example, given two membership functions related to the linguistic concepts “Fast” and “Slow,” a crisp value “5” might be mapped to a set {0.2/Fast + 0.8/Slow} and an interval “[4, 5]” might be mapped to the interval-valued fuzzy set {[0.15, 0.25]/Fast + [0.75, 0.85]/Slow} or to the fuzzy set {0.25/Fast + 0.85/Slow}, depending on the chosen method.

Following this section, a method for ranking the importance of the fuzzy aggregated metrics in relation to the grading problem is presented that allows applying an arbitrary deterministic or random feature selection algorithm to this problem. In short, each imprecise value in the training database will be regarded as a set of possible values. A standard feature selection algorithm will be repeatedly launched over different selections of the sample that comprise possible instantiations of the data. Each selection gives rise to a different ranking, and all of these will be aggregated into a fuzzy membership function, to which a possibilistic meaning is assigned, as described before. Finally, a ranking between these fuzzy memberships will be defined and used to select a relevant subset of features.

### 2.1. Random Feature Selection Extended to Vague Data

In the following, the grades and fuzzy aggregated metrics will be regarded as random and fuzzy random variables, respectively. A fuzzy random variable will be regarded as a nested family of random sets:

(1) $$\begin{array}{c} {\left(\Lambda_{\alpha }\right)}_{\alpha \in (0,1)}, \end{array}$$

each one associated to a confidence level 1 − *α* [13]. A random set is a mapping where the images of the outcomes of the random experiment are crisp sets. A random variable *X* is a selection of a random set Γ when the image of any outcome by *X* is contained in the image of the same outcome by Γ. For a random variable *X* : Ω → **R** and a random set Γ : Ω → *P*(**R**), *X* is a selection of  Γ (written *X* ∈ *S*(Γ)) when

(2) $$\begin{array}{c} X\left(\omega \right)\in \Gamma \left(\omega \right)\;\forall \omega \in \Omega . \end{array}$$

In turn, a random set can be viewed as a family of random variables (its selections.)

Let be *M* + 1 paired samples (*X*_1_^*k*^, *X*_2_^*k*^,…, *X*_*N*_^*k*^) and (*Y*_1_, *Y*_2_,…, *Y*_*N*_), with *k* = 1,…, *M*, from *M* + 1 standard random variables *X* ^1^, *X* ^2^,…, *X* ^*M*^ and *Y*. In this particular case, *M* is the number of metrics and *N* is the number of students. It will be assumed that all universes of discourse are finite. Let be assumed that a feature selection algorithm is a random mapping between the *M* + 1 paired samples and a permutation *σ* of {1,…, *M*} that sorts the metrics according to their relevance:

(3) $$\begin{array}{c} \sigma \left(X_{1}^{1},X_{2}^{1},\ldots ,X_{N}^{M},Y_{1},Y_{2},\ldots ,Y_{N},\omega \right)=\left(\sigma_{1},\ldots ,\sigma_{M}\right)\left(\omega \right), \end{array}$$

where *p*_*ik*_ = *P*(*σ*_*i*_ = *k*) = *P*(*ω*∣*σ*_*i*_(*ω*) = *k*) with *i*, *k* = 1 …, *M*, is the probability that the *k*th random variable *X* ^*k*^ is ranked as the *i*th most relevant feature. If the feature selection criterion is deterministic (e.g., a correlation or mutual information-based criterion [14]) then *p*_*ik*_ ∈ {0,1}. In other cases, successive launches of the feature selection algorithm over the same sample will produce different permutations (think for instance of random forest-based feature importance measures [15]).

Now let *M* + 1 be fuzzy paired samples $\left({\tilde{X}}_{1}^{k},{\tilde{X}}_{2}^{k},\ldots ,{\tilde{X}}_{N}^{k}\right)$ and an also paired crisp sample (*Y*_1_, *Y*_2_,…, *Y*_*N*_) from *M* + 1 fuzzy random variables ${\tilde{X}}^{1},{\tilde{X}}^{2},\ldots ,{\tilde{X}}^{M}$ and the random variable *Y*. Let the list of fuzzy numbers $\tilde{\sigma }=({\tilde{\sigma }}_{1},\ldots ,{\tilde{\sigma }}_{M})$ be defined as

(4) μσ~i(k)=sup⁡{α ∣ P(σi(X11,X21,…,XNM,Y1,Y2,…,YN)=k)≥ϵ    Xik∈S([X~ik]α),  i,k=1,…,M},

for a given small value *ϵ*. It will be shown later in this paper that each fuzzy number $\tilde{\sigma }$ models our incomplete knowledge about the possible ranks of each fuzzy aggregated metric ${\tilde{X}}^{k}$; these metrics will be ordered according to a ranking between fuzzy numbers. In Section 5 a detailed practical case is worked.

## 3. Genetic Learning of Fuzzy Rules from Imprecise Data in Modeling Problems

It was mentioned that an importance-based ranking of features in fuzzy datasets is also uncertain. In the preceding section, a method for dealing with this imprecision was proposed. A secondary problem arises when the automatic grading system has to be learnt from this vague data; however, there exist machine learning algorithms that can be used with this purpose. In this paper, the fuzzy rule learning algorithm NMIC, introduced in [16], will be used. As this is not a widely known method, it is recalled and briefly described in this section for the convenience of the reader.

Fuzzy models, comprising *R* rules with the form

(5) $$\begin{array}{c} If\;\;x\;\;is\;\;A_{r}\;\;then\;\;y\;\;is\;\;B_{r}\;\;with\;\;weight\;\;w_{r}, \end{array}$$

are used, where *x* and *y* are the feature and the output vectors, respectively, and *A*_*r*_ are conjunctions of linguistic labels, which in turn are associated with fuzzy sets. *B*_*r*_ is a singleton. The output $\tilde{Y}$ of the fuzzy model for a fuzzy input $\tilde{X}$ is defined through the membership function

(6) $$\begin{array}{c} \tilde{Y}\left(y\right)=\sup\left\{\tilde{X}\left(x\right)\;|\;Y=\frac{\sum_{r=1}^{R}f_{r}\left(x\right)}{\sum_{r=1}^{R}A_{r}\left(x\right)}\right\}, \end{array}$$

where each function *f*_*r*_(*x*) is a product *β*_*r*_ *A*_*r*_(*x*). *A*_*r*_(*x*) is the membership of *x* to a linguistic expression whose terms are labels of the linguistic variables defined over the input variables, connected by the operators “AND” and “OR.” *β*_*r*_ is the product of the centroid of *B*_*r*_ and the weight *w*_*r*_ assigned to the rule.

### 3.1. A Michigan-Style Genetic Fuzzy Model for Imprecise Data

The NMIC learning method is based on the hypothesis that the best model will comprise rules that are in nondominated sets under confidence and support measures [17]. An extension of the NSGA-II algorithm to fuzzy data is repeatedly launched to obtain Pareto fronts containing nondominated rules in terms of confidence and support. Every front is regarded as a population of a Michigan-type algorithm, where each individual is an antecedent of a fuzzy rule and the whole population is a fuzzy model. Individuals (rules) are weighted and selected by means of a procedure called* SVD select* (explained later in this section). The pseudocode of the NMIC algorithm is shown in Pseudocode 1. The codification and genetic operators are described in [16].

**Pseudocode 1:**
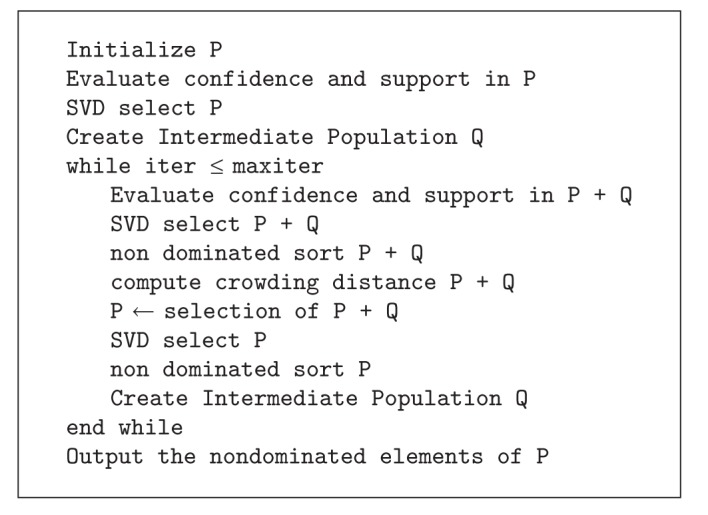
Pseudocode of the NMIC algorithm.

The fitness of an individual has three components: the support of the antecedent of the rule, its precision (dual concept of the confidence in classification), and the weight of the rule. The support is the fuzzy arithmetic-based sum of the memberships of the antecedent for all the points in the sample. The precision is understood as the inverse of the (fuzzy) variance of the examples covered by the rule, that is, all examples in the dataset, weighed by their memberships to the antecedent of the rule.

The following expression is used to compute the precision *p* of a rule (the lower the better):

(7) $$\begin{array}{c} ⨁{\left[{\tilde{Y}}_{n}⊖pA_{m}\left({\tilde{X}}_{n}\right)\right]}^{2}⨂A_{m}\left({\tilde{X}}_{n}\right)\;\exists n\;|\;A_{m}\left({\tilde{X}}_{n}\right)>0\\ ⨁{\left[{\tilde{Y}}_{n}\right]}^{2}\;\text{otherwise}, \end{array}$$

where *p* is the solution to the weighted least squares problem defined by the centers ${\bar{X}}_{n}$ of the data:

(8) $$\begin{array}{c} p=\frac{\sum_{n=1}^{N}Y_{n}A_{m}{\left({\bar{X}}_{n}\right)}^{2}}{\sum_{n=1}^{N}A_{m}{\left({\bar{X}}_{n}\right)}^{3}}. \end{array}$$

Furthermore, observe that the cooperation of the rules only arises if the sum of the fitness values of the individuals is comonotonic with the fitness of the rule base they form. Otherwise, the genetic evolution would not improve the error or the model. The proposed precision and support measures are not enough to achieve cooperation by themselves, as each rule in the population models the part of the space covered by its antecedent, but nothing prevents that more than one rule covers the same area while other areas are uncovered. Therefore, a* SVD select* procedure is introduced that consists in assigning each rule in the Pareto front a weight, with the purpose of obtaining a compact rulebase, while at the same time these weights achieve the best matching between the data and the model.

These weights will be obtained by least squares. Let *A* be the matrix of the memberships of the antecedents of all rules in the Pareto front, at the centerpoint of the inputs. Let *Y* be a column vector with the centers of the desired outputs of the model, and let *W* be another column vector formed by the weights of these rules, those that we want to obtain. The assignment of weights that minimizes the error (and therefore solves the cooperation problem) is

(9) $$\begin{array}{c} K={\left(A^{t}A\right)}^{-1}A^{t}Y \end{array}$$

provided that the rank *r*_*A*_ of *A* coincides with its number of columns, the number of individuals in the Pareto front In most cases, *r*_*A*_ is lower than this; therefore, *C* = *A* ^*t*^ *A* does not have inverse. The common solution to this problem is to apply a singular value decomposition *C* = *UDV* ^*t*^, then cancel the eigenvalues of *D* lower than 10^−6^ times the highest, and take the inverse of the remaining ones, and by last define

(10) $$\begin{array}{c} K=\left[V\cdot \left(\frac{1}{D}\right)\cdot U^{t}\right]A^{t}Y. \end{array}$$

While this assignment solves the cooperation problem, it does not solve the competition problem, because the redundant rules are not discarded. The value of *K* in the preceding equation is that of minimum norm, but what we really need is the definition of the matrix (1/*D*) that produces* the most sparse* definition of *K*, not that with the lowest weights. For example, observe that the definition in (10) will assign the same weight to identical rules, but we want one of them to take all the credit. It is easy to purge the duplicated rules, but it is difficult to remove rules that are (almost) linear combination of others in the Pareto front.

As a matter of fact, the number of individuals we want to assign weights different than zero is the same as the number of not cancelled eigenvalues in *D*. Observe that the columns of the matrix *U* associated with null eigenvalues form a basis of the nullspace of *A* and that means that each individual (each column of *A*), if expressed in the base formed by the columns of *U*, will have at most *r*_*A*_ nonnull coefficients; that is, we will not find more than *r*_*A*_ independent elements in the Pareto front. Therefore, we know that we can set to zero the weights of all the rules but *r*_*A*_. The problem here is how can we determine which columns of *A* will be set to nonzero weight.

The solution is trivial, but computationally intensive. Since the minimum norm is not searched but we are interested in an sparse matrix *K* (thus the number of fuzzy rules is kept as small as possible), in [16] it was proposed not to add a regularization term, that is, not to take the inverse of *A* ^*t*^ *A* + *λI*, but that the eigenvalues of all the submatrices *A*′ of *A* formed by removing only one of its columns are computed. When a submatrix *A*′ whose non null eigenvalues are the same as those of *A* is found, the column is removed and the process restarted from *A*′. At the end of the process, a matrix with *r*_*A*_ columns and full rank is obtained and (9) can be applied.

## 4. Numerical Results

In this section a real-world problem is worked; thus, the benefits of the proposed approach can be compared to the results of alternative approaches. A brief state of the art in software metrics is included first; thus, the experimental setup can be better understood. The experimental design and a description of the field work follow, and the section ends with some compared numerical results.

### 4.1. Software Metrics Used in This Experimentation

As mentioned, software metrics will be used to measure the quality of the students' submitted source code. It will be assumed that better coding leads to higher scoring, but static analysis is also used to obtain additional insights into the student's programs. There are many comprehensive surveys describing the best known software metrics; see for instance [18] or [19]. According to [20], the most relevant software metrics in this context are as follows.

- Number of lines of code: a naive measurement of the code size.
- Ratio between lines of comments and lines of code: a measurement of code documentation.
- Halstead metrics [21]: these metrics have been reported to be useful to evaluate students programs [22].
- Cyclomatic number [23]: often related to* complexity* measures and thus usually referred to as cyclomatic complexity number, but there is not a full agreement about the subject [22]. The cyclomatic number is a graph theory concept that has been translated to software because a program can be modeled as a strongly connected graph [24].

Most of the software analysis tools (http://www.webappsec.org/) provide these features and many other indexes. As the number of software metrics increases, the odds that some of them are closely related or overlap in the property being measured increases too. For instance, it is hard to conceive an increase of the cyclomatic number without a simultaneous increase of the lines of code. Moreover, an increase in the value of a metric is not always consistent with an improvement in the code quality. For instance, it may happen that the complexity of a given problem solution is too low, because the student is unwinding a loop, or the complexity may be greater than expected because the student is using a quadratic algorithm for a problem with linear solution. In either case, the dependence between the metric value and the desired solution is highly problem dependent. Because of this, some researchers propose to use feature selection techniques for finding the best software metrics for the problem at hand. In [25], a stochastic procedure is employed to select the subset of quantitative measures that bring out the best software quality prediction. Another example is [26], where eighteen filter-based feature selection procedures are tested against sixteen software datasets, in this case searching for fault prone modules.

In this paper, a set of software metrics that are commonly used to measure student's source code properties [7] with some additions from [27] for extracting information related with style and structure will be used, and feature selection techniques will be developed that help to select the best set of metrics for each programming concept.

### 4.2. Experimental Design and Field Work

Forty-six volunteering students from the first course of an Engineering Degree in Computer Science at Oviedo University, Spain, participated in this study. The Python programming language was used. Students were allowed to upload as many source code files as they wished, ranging from none to more than a solution for each problem. 800 files were uploaded. Seven programming concepts were studied: Standard I/O, Conditionals, While loop, For loop, Functions, File I/O, and Lists. The evaluation of the students comprised both theoretical and practice skills, with two exams each, at the midterm and at the end of the term. The uploaded exercises were not part of the exams and had no impact on the final grading. 23 software metrics and properties were measured for each source file; thus, the feature selection stage has to choose between 161 different combinations of programming concept and software metric.

The feature selection algorithm to be extended is based on the random forest feature importance measures [15]. A fuzzy rank (see [28]) was used to sort the fuzzy rankings.

The most relevant pairs of found software metric-programming concept are displayed in Table 1. This is the subset for which the best model attained a minimum error; details are given later in this section. Two *α*-cuts of their fuzzy ranks are also given. In Figure 4(b), the whole membership function of the fuzzy rank is plotted for two particular metrics: “COCOMO SLOC” [29] and “McCabe Complexity” [23]. It is remarked that a high degree of overlapping between the memberships of the fuzzy ranks was found in this study.

Observe that the only programming concepts in this subset of metrics are “Conditional” and “File I/O,” meaning that the correlation between the scores and “While loop,” “Functions,” “Lists,” or “Standard I/O” is weaker. This is an unexpected result, since the latter five programming concepts intuitively convey more information about the grading than the two former ones, but this fact can be explained nonetheless if the particular circumstances of this experimentation are taken into account. Students uploaded more exercises at the beginning of the course than at the end; thus, the quality of the information about their capabilities is better for the initial problems. The ranking algorithm has therefore discovered that apparently relevant information may be discarded without lowering the prediction capabilities of the model and suggested to evaluate the performance of a student with the eleven metrics shown in Table 1.

**Figure 2:**
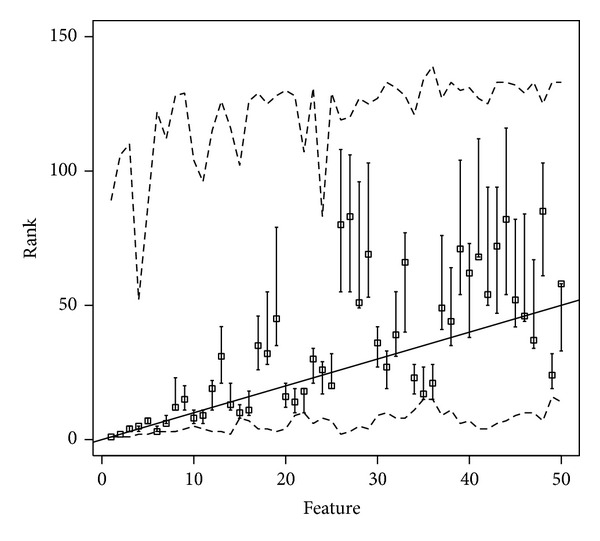
Supports (dashed lines), modal points (bars), fuzzy ranks (abscissa), and crisp ranks (ordinate of the squares) of the 50 most relevant metrics. Those metrics whose square is plotted below the diagonal line occupy a more relevant position under the fuzzy rank.

In Figure 2, the rank of the most relevant metrics, according to the proposed algorithm, is graphically displayed. Supports (dashed lines), modal points (bars), fuzzy ranks (abscissa), and crisp ranks (ordinate of the squares) of the 50 most relevant metrics are displayed. Observe that those metrics whose square is plotted below the diagonal line occupy a more relevant position under the fuzzy rank than they were assigned by the crisp feature selection algorithm. Squares over the diagonal line, on the contrary, are assigned more weight by the crisp algorithm than they are with the fuzzy extension.

**Figure 3:**
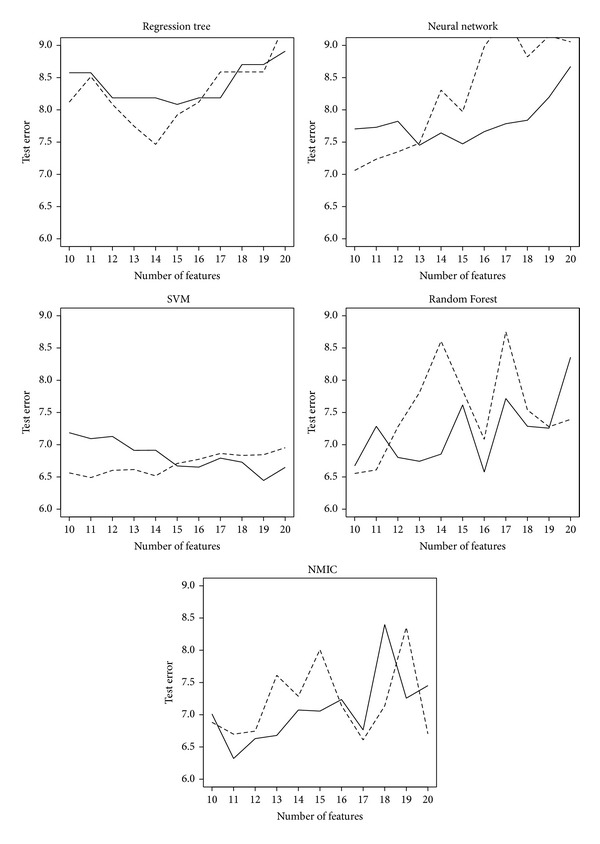
Test errors for feature subsets of sizes 10 to 20. The results associated to a random forest feature importance-based selection, applied to the centerpoints of the fuzzy data, are drawn with dashed lines. The extension of this method to fuzzy data, followed by a learning with the same centerpoints for Regression Trees, Neural Networks, Support Vector Machines, and Random Forest, but the whole fuzzy data for NMIC, are drawn with solid lines.

From a methodological point of view, the proposed technique is robust and the available information is better exploited with the combination of the fuzzy feature selection and NMIC than it is with standard feature selection and model learning algorithms. To prove this fact, regression trees [30], neural networks [31], support vector machines [32], random forests [33], and the NMIC algorithm were launched over subsets sweeping the range between 10 and 20 metrics, found by both the fuzzy extended feature selection algorithm and the original crisp version operating on the centerpoints of the aggregated data. In Table 2 these results are jointly displayed. In Figure 3, test errors corresponding to the selection of the most relevant variables with random forest feature importance measures (applied to the centerpoints of the fuzzy data) are drawn with dashed lines. The proposed extension of the same feature selection method to fuzzy data, followed by a learning with the same centerpoints for Regression Trees, Neural Networks, Support Vector Machines, and Random Forest, but the whole fuzzy data for NMIC, are drawn with solid lines. Observe that if the number of features associated with the lowest test error is chosen as a quality index, the proposed extension improved the accuracy of the grading system in 4 of 5 cases (all but the Regression Tree, with incidentally attained the worst results).

**Figure 4:**
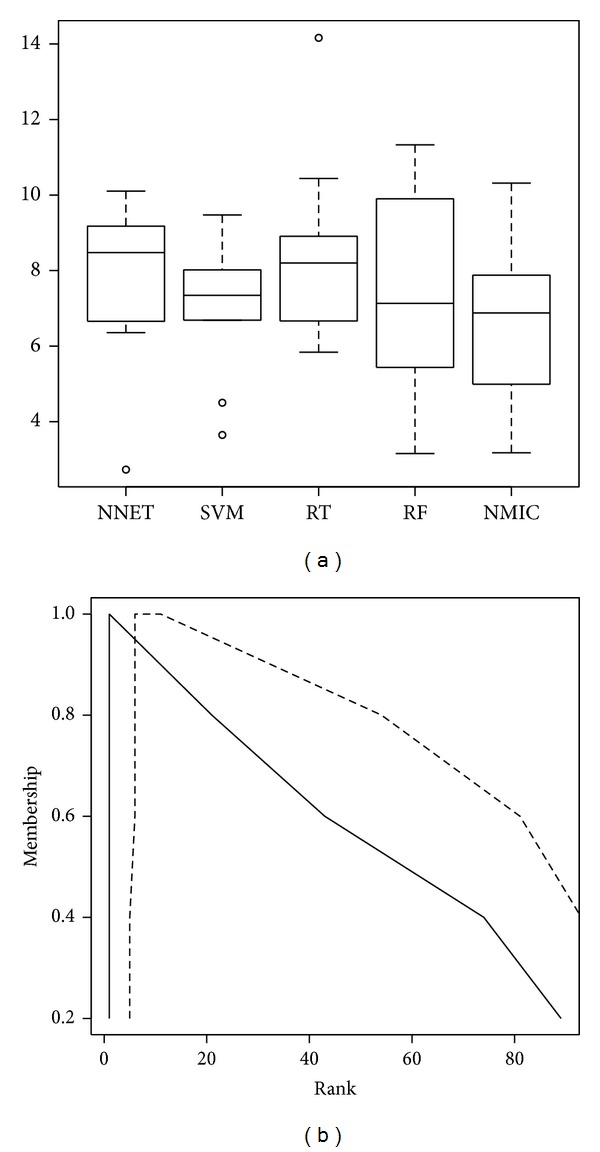
(a) Boxplot showing the statistical differences between the test error of the combination of Neural Networks (NN), Support Vector Machines (SVM), Regression Trees (RT), Random Forests (RF), and NMIC with the feature set computed as described in this paper. NMIC exploits the imprecision in the information better than the alternatives. (b) Membership functions of the ranks of the first (solid) and 10th (dashed) features, that is, COCOMO SLOC and McCabe complexity.

The combination of the NMIC algorithm with fuzzy data was consistently better in all cases (statistically relevant results, according to Friedman/Wilcoxon tests, *P* value better than 0.05). In Figure 4(a) a set of boxplots is drawn, showing the statistical differences between the test error of the combination of Neural Networks (NN), Support Vector Machines (SVM), Regression Trees (RT), Random Forests (RF), and NMIC with the feature set computed as described in this paper. This last graphic is intended to show that NMIC exploits the imprecision in the information better than the alternatives, demonstrating that the fuzzy aggregation loses less information than the alternatives and also that the proposed method is able to exploit this extra information.

### 4.3. Comparison to Other Automatic Grading Systems

The closest to this proposal automatic grading algorithm found in the literature is [7]. In this reference, an online judge/tutor is presented. The purpose of that system is to provide the students of an introductory Computer Programming subject with a quality measure of the submitted code and also with some recommendations to improve it. The students interact with the system via an Eclipse plugin, comparing their solutions against the teacher's ones. The comparison has two parts: first a structural comparison is done; then an evaluation of the correctness using a set of test cases is performed.

This paper is related to the first task, where a fuzzy representation of the algorithm's structure is used. For each exercise the teacher writes his/her best solution and then computes several software metrics (McCabe cyclomatic complexity, COCOMO SLOC, etc.). Three fuzzy sets (Low, Normal, and High) are defined in order to allow certain degree of deviation with increasing penalization as the student's values depart from the teacher's ones. For each software metric and problem, the teacher manually defines each set. Later, the same software metrics are computed for the student's submitted source code. The aggregated membership to teacher's fuzzy sets values is taken as a student's solution quality measurement. The aggregation function has several manually selected weights to adjust the relative importance of the different software metrics for each assignment.

The main two differences between [7] and the method described in this paper are as follows.

1. Only one submission is allowed in [7] for each assignment, and the solution to the problem must be provided by the instructor. In this paper, the metrics of a set of papers can be combined into a fuzzy value, allowing for multiple submissions, missing data, and partially incomplete assignments.
2. The set of metrics is chosen beforehand in [7] but chosen from a pool of metrics in this proposal.

In order to compare both approaches, each of the submitted source codes was measured in five of the seven software metrics proposed in [7]; two of them were not applicable to Python and had to be removed. Some additional changes were effected: since the method in this paper supports using different source files vinculated to the same task, each source file had to be matched with a solution to the problem chosen by hand and also newly coded by the instructor.

**Figure 5:**
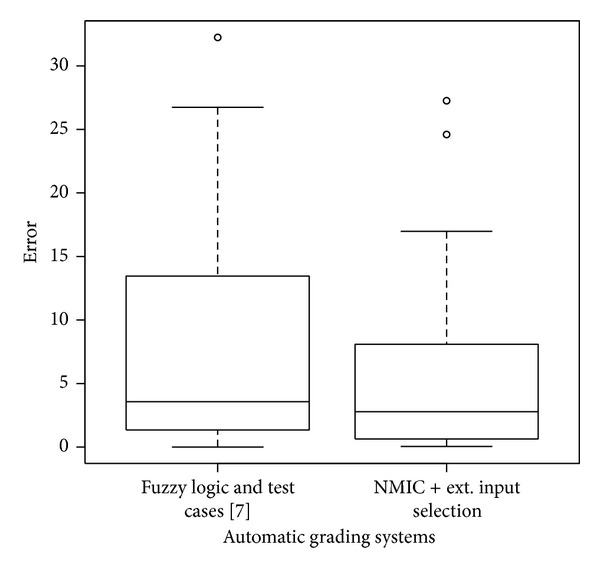
Boxplot comparing the test results (10-cv) of two different automatic grading systems: Fuzzy Logic and Test Cases [7], and NMIC + Extended Input Selection (this paper), showing a statistically relevant difference between both groups.

A 10-cv experimental design was used, whose results are graphically shown in Figure 5. It can be stated that the proposed system is more accurate than [7]. This assert is supported by a statistical test where the hypothesis “both methods produce the same results” was rejected at the 95% confidence level (Wilcoxon test, paired data, *P* value = 0.036, alternative hypothesis: true location shift is greater than 0).

## 5. Concluding Remarks and Future Work

A method for ranking software metrics according to their relevance in an automatic grading system has been proposed. The main innovation of the new method lies in the development of a set of techniques that can make use of a fuzzy aggregation of the information contained in a variable number of exercises about the same learning subject.

From a methodological point of view, the new algorithm is a solid alternative. The combination of a learning algorithm for vague data and the extended feature selection proposed in this paper was shown to make a better use of the imprecision in the information than any of the alternatives, demonstrating that the fuzzy aggregation keeps valuable information and also that the proposed method is able to exploit this. On the other hand, from the point of view of the automated grading techniques, it has been found that the most informative metrics are some measures of the cost and complexity of the code, followed by indicators related to the code size and quality of the documentation. However, there is still a margin for improving this knowledge, as the number of students participating in the study was small and further work is needed to build a larger corpus of hand-graded assignments.

## Figures and Tables

**Table 1:** Most relevant pairs of software metric/programming concept for the field study mentioned in the text. Observe that the programming concepts in this subset of metrics are “Conditional” and “File I/O,” meaning that the correlation between the scores and “While loop,” “Functions,” “Lists,” or “Standard I/O” is weaker.

Programming concept	Description of the metric	Rank 99%	Rank 80%
Conditional	COCOMO SLOC [29]	1 ± 0	11 ± 10
Conditional	Number of tokens	2 ± 0	8.5 ± 7.5
Conditional	Code ratio	4 ± 1	25 ± 24
File I/O	Number of characters	4 ± 1	11 ± 8
Conditional	Number of lines	7 ± 1	21 ± 19
Functions	Number of characters	4 ± 1	43 ± 37
Conditional	Number of keywords	7.5 ± 1.5	36 ± 33
Conditional	Number of comments	17 ± 6	48 ± 44
File I/O	Ratio of comments	17 ± 6	48 ± 44
File I/O	McCabe Complexity [23]	17 ± 9	30 ± 24
File I/O	Number of blocks	17 ± 9	31 ± 25

**Table 2:** Test error or the different regression methods for feature sets ranging from 10 to 20 variables.

Features	Multilayer	SVM	Regression	Random	NMIC
	Perceptron		Tree	Forest	
10	7.703	7.185	8.574	6.671	7.011
11	7.729	7.093	8.574	7.285	** 6.321**
12	7.823	7.128	8.185	6.802	6.629
13	** 7.451**	6.911	8.185	6.742	6.678
14	7.641	6.914	8.185	6.854	7.073
15	7.472	6.670	** 8.084**	7.617	7.056
16	7.661	6.652	8.185	** 6.576**	7.236
17	7.785	6.791	8.185	7.716	6.764
18	7.838	6.728	8.703	7.285	8.399
19	8.195	** 6.445**	8.703	7.256	7.256
20	8.672	6.648	8.909	8.357	7.451
